# The Book World of Medicine and Science

**Published:** 1894-02-17

**Authors:** 


### The Book World of Medicine and Science.

### BOOK REVIEWS.

The Student's Dictionary of Medicine. By Alexander Duane, M.D. (Longmans, Green, and Co., London. Price 21s.)

Dr. Duane, a New York physician, has produced in his "Dictionary of Medicine" a book that will be hailed by students, as it is certainly one of the best of its kind that has appeared for a long while. The author has succeeded in effecting that which he declares to be his object in the preface, namely, to produce a work of really practical utility. He has succeeded in supplying the deficiencies of many a previous dictionary; he has eliminated much that finds its way into such books, and is yet obsolete and useless; and has managed to supply information that is thoroughly up to date. He has left the hackneyed road followed by most compilers, and adopted new methods of arrangement which are distinct improvements. The book is not a Quain; it is purely a dictionary of medical terms, as opposed to one in which medical treatment also finds a place. The clear treatment of these terms is apparent as soon as one opens the book. Reading thus under the heading Abdomen, a distinct description of this part of the human body, its arrangements and functions, are fully explained before side issues on the subject are touched at all; and the throughout simple and practically clear arrangement and explanation is undoubted. One of the best features of the dictionary is the excellent tables which find place in its pages. These are devoted to poisons, their nature and antidote; arteries; canals, and bacteria—these last occupying no less than thirty-five pages to themselves. They are divided to show their origin, morphological character, temperatures favourable to their existence and their properties. Throughout the book all the newest tests and systems are given. The author states that he has endeavoured to meet the difficulty of spelling by adopting that orthography which conforms most closely to analogy and derivation in the words. In this connection we note that he adheres to the old spelling and derivation of the word posthumous, which Hoblyn gives as postumous, from postumus sup. of posterus, coming after. To conclude, we would say that a careful comparison with other dictionaries show that Dr. Duane's long and practical acquaintance with medical terms in connection with dictionaries has enabled him to produce a work which will be both useful and successful.

Pauperism in Great Cities. Its Four Chief Causes.—By Robert Treat Paine, President of the Associated Charities of Boston, &c., author of "Not alms, but a Friend," "How to Repress Pauperism," "Homes for the People," &c. Read at the International Congress of Charities, Correction, and Philanthropy, at Chicago, June 12th, 1893.

Mr. Paine deals with the subject of pauperism as one who realises the magnitude of the problem, and while fully understanding the underlying laws according to which it must be dealt with, does not think these laws must be administered without regard to humanity. Not that he recommends the "humanity" of the cheap philanthropist who would give alms to every beggar, and out-relief to every applicant. He recognises that indiscriminate almsgiving is one of the chief causes of pauperism—the others being foul homes, intoxicating drink, and neglect of child life. With regard to out relief, even when given most justifiably, as in the case of widows with children, Mr. Paine recognises the danger of the family growing up with the pauper taint. But the chief difficulty in treating pauperism is the differentiation of the worthy poor who have fallen on evil days from those who are paupers simply because they choose to belong to "the leisure class among the poor." Mr. Booth's statistics of East London show that there are whole clans that are paupers in grain, the offspring of paupers, themselves paupers from the cradle, and demanding more and more of parochial help as they become the parents of the paupers of a new generation. The like of these are to be repressed; but as Mr. Paine points out, mere repression fails to solve the problem. Even the old heroic Tudor method of hanging all and sundry as rogues and vagabonds did not drive pauperism from the land, and at present there are in all our great cities people who, under existing conditions, have no chance of attaining to any self-respecting life. Mere repression brutalises them; living as they do they cannot be other than they are. To furnish them with homes where social decencies are possible is to give them at least the option of a better life. Some will not take it; they prefer to pay as much for a mouldy cellar as for a decent flat because "they love the darkness rather than the light," but all are not of that temper. But good homes alone will not cure pauperism, at the back of which lies inefficiency—an incompetence to do work which can claim a decent wage. The machinery of fixing a minimum wage cannot help these; there is work which society must have cheap, or it will do without it altogether. Mr. Paine quotes a thoughtful remark from an essay on the "Ethics of Social Progress," by Professor Franklin H. Giddings. "Neither oppression nor greed has been at any time the first cause of legal bondage or of economic dependence. . . . . We are told incessantly that unskilled labour creates the wealth of the world. It would be nearer the truth to say that large classes of unskilled labour hardly create their own subsistence." Clearly, then, the first thing to do is to give those who are young enough to learn such a training, general and technical, as will help them to climb out of the class of the unskilled. To do so will profit society as well as the else predestined paupers. But there still remain those who, having done their best, have fallen by the way. The law, like the rain, must deal in the same fashion with the deserving and the undeserving, but private charity has the right to discriminate. Of course, this discrimination must be founded on knowledge, and must not dwindle into mere almsgiving. The friendly visitors of the Associated Charities of Boston are forbidden to give alms on their own responsibility to any family they visit. The efforts of the association are devoted, not to maintaining paupers, but to taking them out of pauperism. The same method has been pursued successfully in the work of the North Kensington Friendly Visitors. It remains to be seen if it will answer as well on a larger scale. To students of the question this little book of Mr. Paine's should be both useful and encouraging.

Kelly's London Medical Directory. (Kelly and Co., London.)

The Directory for 1894 follows the way of most of these books, and appears in enlarged form. Last year the list of physicians and surgeons occupied 283 pages, this year it has filled 307 pages. The volume for 1894 displays the usual completeness which renders it an almost indispensable addition to the library of members of the medical profession, institutions, and people of affairs. The arrangement is simple, and convenient for reference, the only improvement we could suggest being that the street directory should directly follow the names of the profession, thus keeping the purely medical sections together. The list of nursing institutions will be found to be complete and reliable. This list, together with that of medical publications and of appliance makers, help to make Kelly's Annual a more handy and comprehensive volume for the use of Londoners than its more pretentious rivals.

